# Microscale generation and control of nanosecond light by light in a liquid crystal

**DOI:** 10.1038/s41566-025-01693-2

**Published:** 2025-06-03

**Authors:** Mahendran Vellaichamy, Uroš Jagodič, Jaka Pišljar, Jaka Zaplotnik, Urban Mur, Andreja Jelen, Andriy Nych, Deepshika Malkar, Anna V. Ryzhkova, Miha Škarabot, Miha Ravnik, Igor Muševič

**Affiliations:** 1 Condensed Matter Department, J. Stefan Institute, Ljubljana, Slovenia; 2 Faculty of Mathematics and Physics, University of Ljubljana, Ljubljana, Slovenia; 3 Department of Engineering Science, University of Oxford, Oxford, UK; 4 Department of Molecular Photoelectronics, Institute of Physics, Kyiv, Ukraine

**Keywords:** Liquid crystals, Liquid crystals

## Abstract

The softness of liquid crystals, their anisotropic material properties, their strong response to external fields and their ability to align on patterned surfaces makes them unsurpassable for a number of photonic applications, such as flat-panel displays, light modulators, tunable filters, entangled photon light sources, lasers and many others. However, the microscale integration of liquid crystals into microphotonic devices that not only perform like silicon photonic chips but also use less energy, operate exclusively on light, are biocompatible and can self-assemble has not been explored. Here we demonstrate a soft-matter photonic chip that integrates tunable liquid-crystal microlasers and laser microprinted polymer waveguides. We demonstrate the control of the liquid crystal’s microlaser emission by nanosecond optical pulses and introduce the concept of resonant stimulated-emission depletion to switch the light by light. This opens a way to design an entirely new class of photonic integrated devices that can be made both biodegradable and biocompatible with a rich variety of applications in medicine, wearable photonics and logic circuits. We anticipate that soft-matter photonic circuits will not only outperform solid-state photonics in terms of a huge reduction in the number of production steps, the use of non-toxic chemicals and a better energy efficiency, but also could open an avenue to the paradigm of soft-matter photonics.

## Abstract (web summary)

Combining advanced photonics with reconfigurable liquid crystalline self-assembled structures allows control of a liquid crystal’s microlaser emission by nanosecond optical pulses and the ability to switch off the laser emission from the liquid crystal using the resonant stimulated-emission depletion process, providing a design for a new class of photonic integrated devices.

## Main

The dual nature of liquid crystals (LCs)^1^, which combines the anisotropic material properties of solids with the fluidity of liquids, makes them unsurpassable for a number of photonic applications^2–6^. In flat-panel displays, light modulators and tunable optical filters, LC molecules respond via their dielectric properties to very low electric fields and thus modulate the intensity of transmitted or reflected light^3^. Distributed-feedback LC lasers^4,5^ are made of chiral LCs with fluorescent dyes, to emit nanosecond light pulses with wavelengths that can be tuned over a large range. While solid-state distributed-feedback lasers require a number of processing steps, an LC distributed-feedback laser is self-assembled in a single stroke and in a fraction of a second, clearly highlighting the advantages of LCs over solids. Recently, the huge optical nonlinearities of ferroelectric nematic LCs were exploited to generate tunable, entangled photon pairs through a parametric down conversion in soft matter^6^.

While the physical size of commercial LC devices is in the metre to millimetre range^2,3^, little effort has been devoted to the engineering and integration of LC devices on the microscale, which is mainly due to the limitations of engineering soft matter at the nanoscale. Recently, direct laser writing (DLW)^7^ has emerged as a powerful tool to produce microscale polymer structures with ∼100 nm resolution. DLW has been used to print polymer waveguides coupled to polymer–LC microcavities that can be tuned by light^8^, to create complex LC mesostructures for light modulation^9–13^, to produce complex colloids, such as colloidal knots and helices^14,15^, and to study fundamental topological phenomena in LCs. While this indicates the possibility to design polymer–LC microstructures akin to solid-state photonic integrated circuits, light generation^16^ and control^17,18^ on a microscale remains largely unexplored in such structures.

Contemporary photonic platforms generally make use of silicon^19,20^ to control the flow of near-infrared photons using electro-optic effects, where the index of refraction is varied by an electric field^21^ or light^22^. Because the silicon cannot emit light efficiently, heterogeneous integration of III–V microlasers is needed to illuminate silicon photonic integrated circuits to enable advanced complementary metal–oxide–semiconductor processing at high volume with low cost and high yield. Mainstream solid-state photonic platforms, which are likely to revolutionize data streaming in the near future, even though they are based on phenomena that were discovered nearly half a century ago, are energy consuming, use hazardous chemicals, require precious metals and produce large amounts of dangerous waste^23,24^. To generate and control the flow of nanosecond light pulses by light in microscale soft matter that is biodegradable, biocompatible and uses less production energy is therefore a major challenge.

Here we demonstrate a soft-matter photonic approach that exploits the room-temperature self-organization of soft organic matter to develop functionally integrated photonic devices and can be made biodegradable, tunable and wearable. The light is guided by DLW-printed polymer waveguides, while the laser light is generated by self-assembled LC microlasers and modulated by light using stimulated-emission depletion (STED)^25^ in LC microresonators. Microlasers made from cholesteric LCs (CLCs) have been demonstrated with the smallest resonator cavity of ∼2 μm^2^ × 8 μm that requires a pumping fluence of ∼80–300 pJ μm^−2^ to generate single, nanosecond optical pulses in the visible region. A fraction of this fluence, that is, ∼5 pJ μm^−2^, is needed to switch off this microlaser by another laser pulse. This photonic platform has the potential to outperform solid-state photonic platforms in terms of the self-assembly of critical photonic elements—microlasers—which not only reduces the number of production steps by orders of magnitude but also can use biocompatible materials and be ecofriendly.

## Results and discussion

**Fig. 1:**
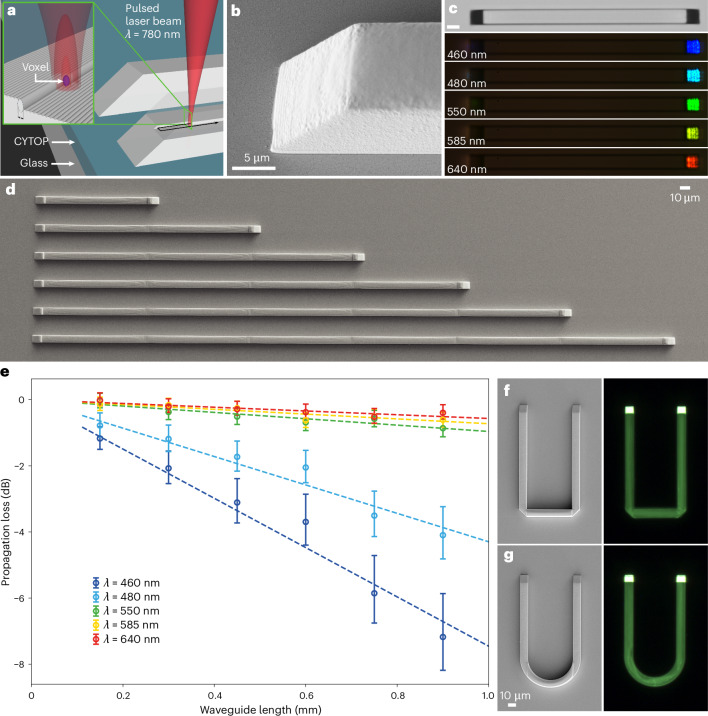
Polymer photonic waveguides are printed on CYTOP on glass in any form and shape, using printed microprisms for the efficient coupling of visible light in and out of the waveguides. **a**, Illustration of laser printing of waveguides and microprisms using two-photon polymerization of resin on an underlayer of the low-refractive-index (*n* = 1.33) polymer CYTOP to optically isolate the waveguide from the glass. **b**, SEM image of a microprism that provides coupling of the light in and out of the waveguides with a insertion losses of 1.1 dB. **c**, The entire visible spectrum of light can be transmitted through IPS resin waveguides (Methods). Scale bar, 10 μm. **d**, SEM image of DLW-printed IPS waveguides on CYTOP/glass of different lengths. **e**, Propagation losses of 10 μm × 10 μm cross-section waveguides for various wavelengths (*λ*). Each point represents the mean propagation loss determined from three repeated measurements on a waveguide of particular length and wavelength. Error bars represent the standard deviation of the determined values. **f**, SEM (left panel) and optical microscope images (right panel) of a U-shaped waveguide with two right-angle prisms in the corners. An additional ∼0.5 dB loss is due to the reflection from each corner microprism. **g**, In half-circular-shaped waveguides the additional losses due to the 35 μm radius of curvature are 1.9 dB. A fluorescent dye was added to the polymer to visualize light propagation in right panels of (**f**) and (**g**).

**Extended Data Fig. 1:**
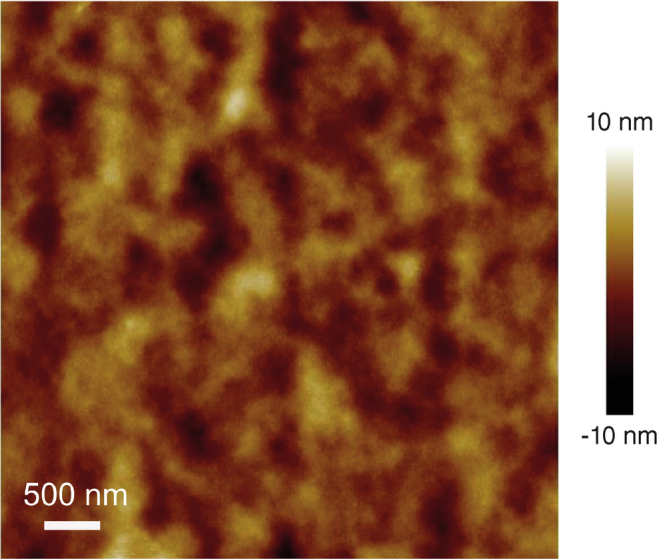
Topography of the DLW printed waveguide’s surface. The RMS surface roughness of this 5 × 5 μm area is 1.8 nm.

Modern photonic platforms, such as silicon or silicon nitride, use total internal reflection from the surface of a strip of high-refractive-index material to guide light along the ∼300-nm core of the waveguide with very low losses of less than ∼0.1 dB cm^−1^. While silicon waveguides are usually produced lithographically, we printed the polymer waveguides directly using DLW^7^. A tightly focused spot of an infrared laser is moved in a photosensitive polymer, leaving behind a line of photo-polymerized resin (Supplementary Video 1). The polymerized voxel is very narrow, that is, ∼200 nm, because the polymerization takes place in a two-photon absorption process, which implies a shorter wavelength and tighter foci. The waveguides are printed on top of a thin layer of low-refractive-index polymer (CYTOP) using a photosensitive resin (IPS), as shown in Fig. 1a. The propagation losses of the waveguides are measured by coupling the light from a tunable laser into the waveguide using total reflection from the right-angle microprism, which is an integral part of the waveguide (Fig. 1b). After travelling over a distance *L*, the light is reflected from the second microprism and out of the waveguide to the detector (Fig. 1c). Several waveguides of different lengths *L* and identical cross-section are printed (Fig. 1d), and for each length the output intensity of the light is measured (Supplementary Video 2). This gives the light intensity as a function of the propagated distance, shown in Fig. 1e. While the propagation losses are substantial for blue light (Fig. 1e), the losses are very low for the red part of the spectrum, that is, 5.7±1.5 dB cm^−1^, mostly due to light absorption in the resin^26^. The root mean square (RMS) surface roughness of the as-printed waveguides is ∼2–3 nm, as measured with an atomic force microscope, and the coupling loss of each microprism is 1.1 dB (Methods and Extended Fig. 1). Compared with straight waveguides of the same length, the rectangular-bent waveguide (Fig. 1f) exhibits an additional ∼1.1 dB loss due to the two 90° turns, and the waveguide with a 35-μm-radius semi-circular section in Fig. 1g shows an extra ∼1.9 dB loss.

**Fig. 2:**
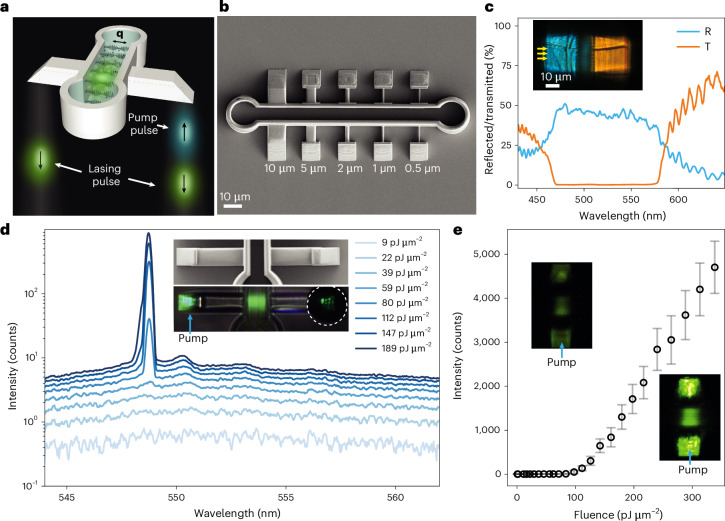
DLW-printed CLC microlaser. **a**, A CLC laser is made by confining the helical structure of a CLC (grey ellipses indicate orientation of CLC molecules; **q** indicates helical wavevector), doped with 0.2 wt% of fluorescent molecules in a DLW-printed cavity with an ∼8–12 μm gap. The blue-light pulses excite the fluorescence, which is amplified by the stimulated emission of light bouncing back and forth along the periodic helix, finally resulting in the lasing of green light. **b**, SEM image of an IPS polymer 8.2 μm cavity that accommodates 5 microlasers, each connected to coupling microprisms with IPS waveguides of 10 μm, 5 μm, 2 μm, 1 μm and 500 nm thickness. **c**, Spectrum of RHC-polarized light reflected (R) and transmitted (T) through the CLC in a scaffold. Inset: microscope image of IPS scaffold with CLC that is illuminated with RHC-polarized white light. The blue light is reflected from the CLC; the red light is transmitted. **d**, The emission spectra of an 8.2 μm microlaser at different pump fluences. The amplified spontaneous emission at intermediate pump levels is followed by a transition to lasing at a fluence of ∼80 pJ μm^−2^. Inset: details of a scaffold hosting a CLC laser with 10 μm waveguide and 2 prisms. Light is collected via circular pinhole (dotted circle). **e**, Lasing threshold of an 8.2 μm microlaser. Each point represents the mean lasing peak intensity averaged over 100 pulses; error bars show its standard deviation. Insets: the light emitted from the cavity, below (15 pJ μm^−2^) and above (150 pJ μm^−2^), the threshold for lasing.

We generate the laser light from an ∼8 μm layer of a CLC that is confined to a DLW-printed microscaffold (Fig. 2a,b and Supplementary Video 3) and integrated with waveguides. The CLC consists of chiral LC molecules that are locally aligned with each other, but average molecular orientation spontaneously twists in space to form a one-dimensional helical structure with wavevector **q**. This structure exhibits a stop band in the reflection spectrum, reflecting circularly polarized light of the same handedness as the helix. The width and the position of the reflection band depend on the indices of refraction of the CLC and the helical pitch, and are tunable. When an optical gain material, for example, fluorescent-dye molecules, is dissolved in the CLC, the CLC layer will emit laser light when the dye molecules are excited with a sufficiently strong, external light pulse^4,5^. The CLC emits tunable laser light at the wavelength of the band-edge mode that represents slow light, bouncing back and forth within the periodic birefringent CLC structure.

A CLC microlaser is produced by injecting a dye-doped LC into an IPS polymer scaffold that was DLW-printed on a 500 nm CYTOP layer on glass (Methods). Figure 2b shows a scanning electron microscopy (SEM) image of the polymer scaffold that forms an 8.2 μm × 100 μm × 10 μm cavity connected to 5 pairs of optical waveguides of different cross-sections with right-angle prisms to couple the light into and out of the cavity. A cavity gap of 8.2 μm was chosen for optimum lasing efficiency. Using a micro-injector, the cavity is filled (Supplementary Video 4) with GCHC10146 LC that was doped with a 3.74 wt% chiral dopant R5011. This CLC has a high birefringence of Δ*n* ≈ 0.405 that produces an ∼120-nm-wide stop band with lasing-edge mode R1 at ∼550–570 nm. We have chosen this high-birefringence CLC because it provides a higher *Q*-factor of the CLC distributed-feedback cavity and a lower lasing threshold. After white light from a supercontinuum laser is sent through the microprism to the CLC sandwiched inside the cavity, we can see that blue light is reflected from the CLC and red light is transmitted through the CLC, as shown in the inset in Fig. 2c. This confirms the very good molecular alignment of the CLC in the polymer cavity, which can be explained by the formation of nanogrooves due to the interference of the DLW writing beam with the reflected beam^10^. The cavity filled with this CLC strongly reflects the right-handed circularly (RHC) polarized light in the wavelength window between 470 nm and 570 nm, as shown in Fig. 2c. In the next step, we dope the same CLC mixture with 0.2 wt% of PM580 fluorescent dye and inject it into the DLW-printed optical cavity, similar to Fig. 2b. The dye provides a uniformly distributed optical gain within the CLC Bragg optical microcavity, which is needed for lasing, and shows good photostability and optical gain^27^.

**Extended Data Fig. 2:**
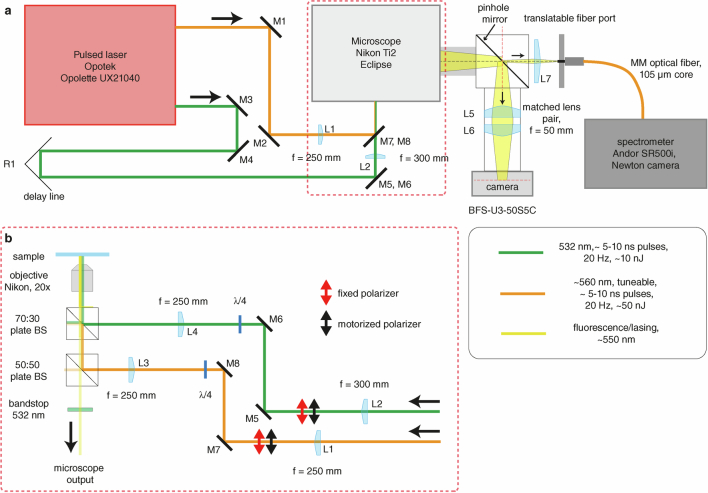
Optical and microscopy setup. **a**, Schematic of the optical setup used for experiments on DLW printed microlasers. The components are mounted on the optical table. At a repetition rate of 20 Hz pulses of a 532 nm pump and a tuneable STED beam exit the laser and are steered using mirrors (M) and shaped using lenses (L). The right hand side of the schematic depicts the xyz translatable detection part of the experiment. Light that is transmitted through the pinhole mirror is collected by a multimode optical fibre, while light reflected from the pinhole mirror is collected by the camera. **b**, A detailed view of the part marked with a red-dashed rectangle around and within the microscope.

The lasing from the CLC microcavities is excited using a linearly polarized 532 nm, nanosecond, externally pulsed laser with s-polarization, which is focused on the microprism, hitting the CLC with polarization that is parallel to the director at the input CLC surface (Methods and Extended Data Fig. 2). By increasing the pulse energy of the pump, we first observe a broad fluorescence background that develops into a broad peak, when the pulse of the pump is just strong enough to initiate the lasing, as shown in Fig. 2d. The broad peak is due to the amplified spontaneous emission and is a precursor of a much narrower lasing line that emerges immediately after the lasing threshold is crossed (Fig. 2d and Supplementary Fig. 1). As shown in the lasing-threshold curve in Fig. 2e, a 10 μm × 10 μm footprint laser starts lasing at an ∼80 pJ μm^−2^ fluence (see Methods for the definition of beam diameter), which corresponds to an ∼2.5 nJ pulse energy. The onset of the lasing is also seen by the appearance of characteristic speckles of random interference, clearly visible in the image of the lasing cavity in the inset of Fig. 2e, right panel. The lasing line is centred at the position of the first R1 eigenmode of the cavity, which is at the red edge of the CLC stop band^28^. We never observed lasing from the R2 mode, which has much lower *Q*-factor and does not sustain lasing^28^.

**Fig. 3:**
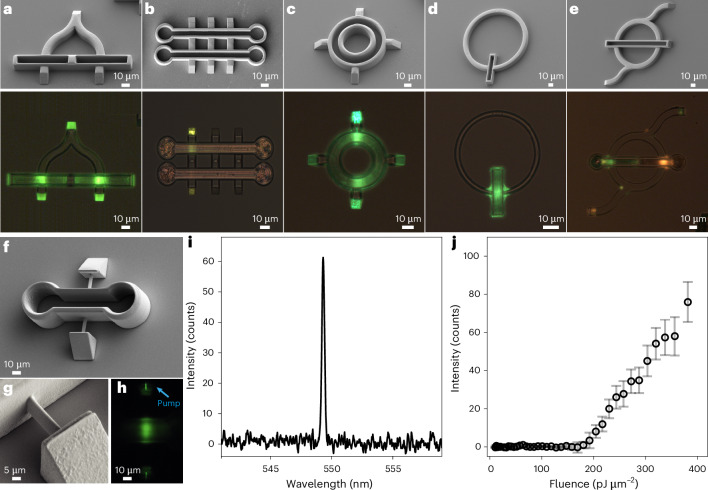
Different design and geometry of CLC microlasers and the smallest operating CLC microlaser. **a**, Two CLC cavities are excited to lasing in parallel. **b**, Two different CLC microlasers are cascaded. **c**, In an annular CLC microlaser, the CLC forms a closed ∼10 μm ring. **d**, A ring CLC microlaser with a single cavity. **e**, A ring microlaser with two coupled cavities. The images in the top row are SEM images, the images in the bottom row are optical microscope images. **f**, The smallest CLC microlaser has a 8.2-μm-long cavity filled with high-birefringence CLC and is pumped via 1 μm waveguides. **g**, Detail of a 1 μm waveguide connecting a 10 μm right-angle prism and the cavity. **h**, The smallest microlaser in operation, laser emission is seen from two small green spots on the top and bottom prisms. **i**, Lasing spectrum of a 1 μm waveguide CLC microlaser. **j**, Lasing threshold of the 1 μm thickness waveguide CLC microlaser shown in **f**–**h**. Each point represents the mean lasing peak intensity averaged over 100 pulses; error bars show its standard deviation.

The soft nature of the LCs and the inherent ability of DLW to print in three dimensions makes it possible to fabricate a number of CLC lasing devices with different design and geometry, as presented in Fig. 3a–f. Lasing can be excited in parallel (Fig. 3a) or serially (Fig. 3b) connected CLC cavities, emitting tunable and distinct wavelengths. Because the CLCs are fluid, an annular laser cavity can be made with a ring-like CLC cavity that is hosting a number of coupling prisms (Fig. 3c). A ring microlaser with single or multiple coupled laser cavities can be made, as shown in Fig. 3d,e. The smallest operable CLC microlaser that we made is shown in Fig. 3f–h (Supplementary Videos 5 and 6). The cavity length is 8.2 μm and the laser is excited via a polymer waveguide with a cross-section of ∼2–3 μm^2^. The spectrum of the emitted light shows a single mode (Fig. 3i and Supplementary Video 7) and the lasing-threshold fluence is somewhat higher (Fig. 3j and Supplementary Video 8).

**Fig. 4:**
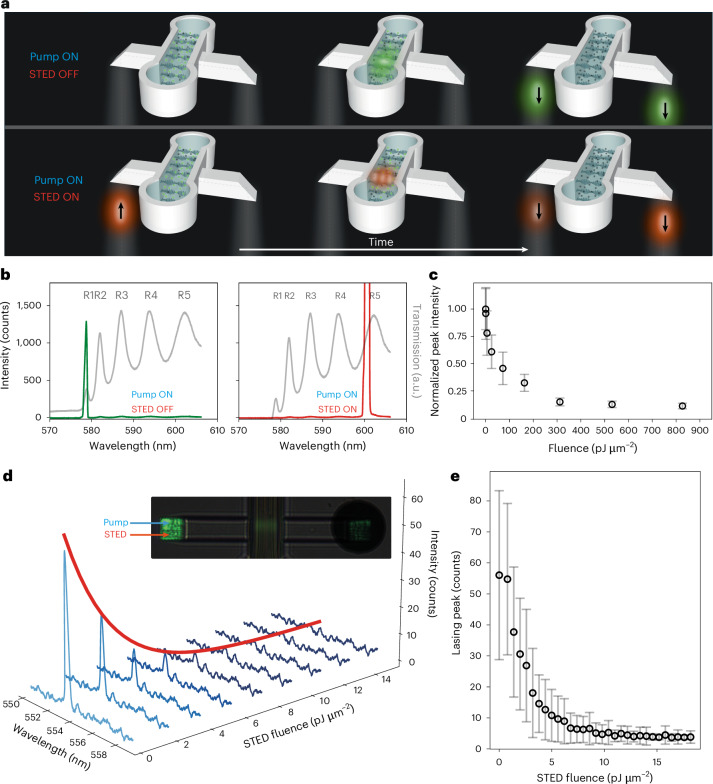
Control of lasing with resonant STED in CLC microcavities. **a**, Illustration of lasing depletion by resonant STED. After excitation, the CLC microlaser will emit two laser pulses of nanosecond duration, propagating in opposite directions of the helix (green blobs, 578 nm). However, if the excited CLC is illuminated with a nanosecond STED pulse of 601 nm (orange) within the time delay *τ* < 1 ns, the STED pulse resonantly takes the stored energy, depletes the 578 nm lasing and stimulates lasing at STED 601 nm. **b**, Depletion of the 578 nm lasing (green curve) by the 601 nm STED pulse (red curve) in a bulk CLC sample of thickness 8.2 μm. The continuous grey curve is the transmission of the CLC. **c**, The efficiency of the resonant STED in bulk CLC. The normalized intensity of the 578 nm lasing peak as a function of STED fluence. **d**, Resonant STED in a CLC microcavity. Spectra are showing the lasing peak (553.5 nm, slightly different from **b** and STED peak (560 nm) taken at different fluences of the STED pulse. Inset: both pump (532 nm) and STED (560 nm) pulses are sent to the CLC cavity via a 10 μm square polymer waveguide. The black circle on the right is the pinhole that collects the light. **e**, The lasing peak (553.5 nm) as a function of the STED fluence. Each point in **c** and **e** represents the mean lasing peak intensity averaged over 100 pulses; error bars show its standard deviation.

Here we show how a beam from an integrated CLC microlaser can be switched on and off by another laser using STED^25,29,30^ in resonant cavities, as illustrated in Fig. 4a and Supplementary Video 9. Consider a fluorescently labelled CLC in a microcavity, as shown in the upper sequence of images in Fig. 4a, that has been excited by a strong pump beam above the lasing threshold (excitation on, STED off). In a fraction of time, the microlaser will emit laser pulse of nanosecond duration, propagating in opposite directions along the helix (green blobs). However, if before spontaneous lasing in green, another (STED) pulse (redshifted to orange) is sent through the cavity (lower sequence of images in Fig. 4a), the green lasing will be depleted, because the number of STED photons will be amplified via stimulated emission inside the resonant cavity and no energy will be left for lasing. This suggests that light signals could be controlled by light via stimulated emission in optical cavities.

We tested this idea by first demonstrating resonant STED in ∼10-μm thick cells of CLC, doped with fluorescent dye, with CLC molecules anchored parallel to the surfaces of the cell. Figure 4b shows the transmission spectrum of such a CLC cell for the RHC-polarized light (the same handedness as the CLC) in the vicinity of the photonic bandgap. For wavelengths shorter than ∼580 nm, the transmission is low due to the strong Bragg reflection of the circularly polarized light, with a series of transmission oscillations. The maxima of these oscillations (that is, R1, R2, R3 and so on) correspond to the wavelengths of the standing light modes^28^ in a layer of CLC with a thickness *d*. After the fluorescent CLC is excited with a linearly polarized, ∼1,000 pJ μm^−2^ light pulse of wavelength 532 nm, it emits laser light near the position of the R1 mode, at ∼578 nm (Fig. 4b, left). However, if we illuminate the excited fluorescent CLC with a linearly polarized STED pulse with an ∼601-nm wavelength within 1 ns of the 532 nm excitation pulse, the CLC lasing is heavily suppressed (Fig. 4b, right). This 601 nm STED wavelength is close to R5 resonant mode indicated in Fig. 4b, and could not be shifted to the more efficient R2 mode because of experimental limitations due to dichroic mirrors. As shown in Fig. 4c, the fluence of the STED pulse that is needed to suppress the CLC lasing by 10 dB (90%) is exceptionally small, that is, of the same order of magnitude as the excitation pulse fluence, that is, ∼300–400 pJ μm^−2^, a major difference to typical STED used in super-resolution imaging, where much larger energy is needed to deplete the fluorescence.

In conventional STED imaging, the energy needed to deplete the excited states of the fluorescent molecules is at least three orders of magnitude larger than the excitation pulse^31^. This is due to the single pass of the STED photons through the specimen with excited fluorescent-dye molecules and a consequent small probability of depletion. In contrast, when the STED photons enter a cavity that is in resonance with their frequency, the photons are resonantly bouncing back and forth inside the cavity. This effectively slows down the photons, decreases their group velocity and increases the time that the STED photons spend inside the cavity. As a result, the interaction of the STED photons with the excited states of the fluorescent molecules is strongly enhanced due to the multi-pass nature of the photons in the resonant cavity; therefore, increasing the cross-section for the STED effect by the *Q*-factor of the cavity.

We next demonstrate (Fig. 4d) the suppression of lasing from the integrated CLC microcavities using resonant STED (Supplementary Videos 10 and 11). Note that the CLC in these microcavities has a slightly different composition and the R1 lasing mode is at ∼553 nm, which is different from the R1 mode at ∼578 nm, studied in bulk CLC lasers. The 532 nm linearly polarized excitation beam is focused on the base of the 10 μm microprism (s-polarization) and is guided into the 8.2-μm-gap microcavity with the CLC (inset to Fig. 4d). The light emitted from the CLC structure is collected with a pinhole (dark circle in the inset to Fig. 4d, right) and spectrally analysed. The 560 nm STED pulse is time-synchronized with the 532 nm pulse, which is focused on the same entrance prism, ensuring good spatial overlap of both beams. Owing to experimental limitations (532 nm blocking filters), STED could not be tuned to the more efficient R2 mode. Figure 4d shows the spectra of light emitted from the CLC microcavity at different single-STED-pulse energies. At zero STED fluence, the microlaser emits pulses at 553.5 nm with a count rate of ∼60 photons per pulse. However, when the STED fluence is slightly increased, it is clear that the lasing signal at 553.5 nm is very effectively suppressed. Surprisingly weak STED fluences of ∼5–10 pJ μm^−2^ are needed to completely suppress the lasing from the microlaser just above the lasing threshold, as shown in Fig. 4e.

**Fig. 5:**
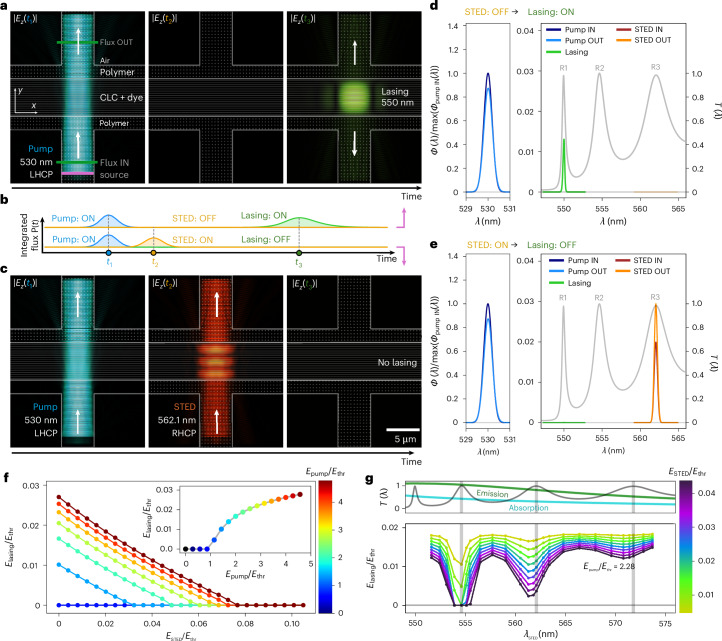
Numerical simulations of lasing and STED in waveguide and CLC cavity. **a**, Time sequence of electric field |*E*_*z*_(*x*, *y*, *t*)| in the lasing regime. Lasing is induced by a left-handed circularly polarized (LHCP) pump pulse. The thick white lines are the boundaries between the materials, the empty texture represents the air, the thin horizontal lines are CLC with dye, and the dotted texture represents the cavity walls and waveguides. **b**, Time-dependent integrated flux measured at the ‘flux OUT’ plane for the lasing and STED regimes. The flux of the 530 nm pump pulse is in blue; orange corresponds to the 562.1 nm STED pulse; and green to the 550.0 nm lasing pulse (Supplementary Video 10). For visualization, the flux of STED versus pump pulse is multiplied by a factor of 25 and the lasing pulse by a factor of 200. **c**, Time sequence of electric field |*E*_*z*_(*x*, *y*, *t*)| in the STED regime (Supplementary Video 10). A right-handed circularly polarized (RHCP) STED pulse, applied after the pump pulse, suppresses lasing. **d**, Spectrum of pulses accumulated at the ‘flux IN’ and ‘flux OUT’ areas over the simulation time in lasing. All spectra are normalized to the maximum of the pump pulse at ‘flux IN’. **e**, Energy spectrum of pulses at the ‘flux IN’ and ‘flux OUT’ over the duration of the pulse sequence in STED regime. **f**, Lasing pulse energy as a function of the energy of the STED pulse with a wavelength of the R3 mode (562.1 nm) for different values of the pump-pulse energy. Inset: lasing energy as a function of the pump-pulse energy in the absence of the STED pulse. All energies are scaled to the lasing-threshold energy of the incoming pump pulse. **g**, Lasing energy as a function of the wavelength of the STED pulse for selected values of the STED energy and at a fixed pump energy *E*_pump_/*E*_thr_ = 2.28, compared with the transmittance of the CLC, emission and absorption spectra.

**Extended Data Fig. 3:**
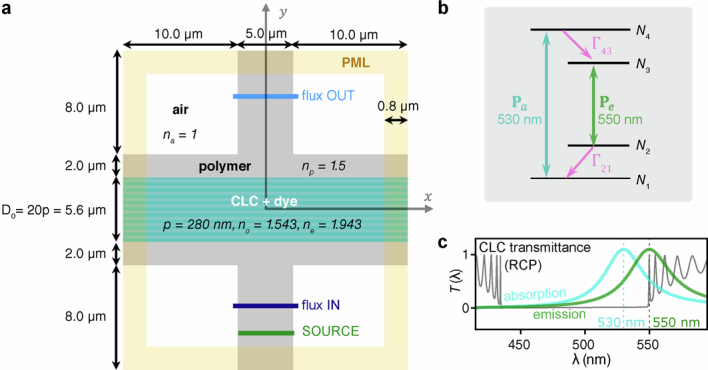
Schematic representation of simulated photonic microstructure and dye in numerical simulations. **a**, 2D simulation geometry: Right-handed cholesteric liquid crystal (CLC) doped with a 4-level dye is confined between polymer walls which further extend into waveguides. **b**, Schematic representation of energy levels in a 4-level saturable gain material. **c**, Transmittance of the CLC for RHCP light, and absorption and emission spectra of the gain material.

The lasing and its suppression by STED in the CLC microstructures are modelled with full Maxwell’s equations using the finite-difference time-domain method^32,33^, extended for light absorption and stimulated emission via a continuum model of saturable, multilevel optical gain. The simulation geometry is shown in Fig. 5a,c with a marked area of the radiation source and two cross-sections (IN and OUT), where the Poynting flux is measured (Methods and Extended Data Fig. 3). The right-handed CLC with dispersed four-level dye molecules and a helical axis in the *y* direction is confined between two polymer walls, which further extend into the waveguides on both sides. At the beginning of each simulation, the dye is in the ground state.

**Extended Data Fig. 4:**
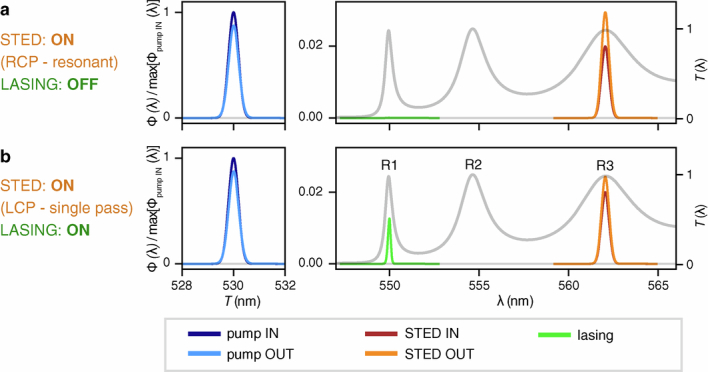
Comparison of resonant and non-resonant STED. **a**, Spectra of pump, STED and lasing pulses accumulated at “flux IN” and “flux OUT” for RHCP STED pulse. **b**, Spectra of pump, STED and lasing pulses accumulated at “flux IN” and “flux OUT” for LHCP STED pulse.

A pump pulse of left-handed circularly polarized (opposite handedness to the right-handed CLC helix) light at the 530 nm absorbance maximum of the dye is sent through the CLC and is partially absorbed, which locally excites the dyes (Supplementary Video 12). Lasing evolves and amplifies from the initial electric-field fluctuations via stimulated emission from the excited dye. It occurs as the first resonant mode R1 (that is, the one with the highest *Q*-factor) that is located at 550 nm, that is, on the red side of the bandgap^28^. The lasing is amplified until there is enough optical gain available and then gradually fades out, when dye is depleted. Thus, the overall output of the CLC is a single laser pulse at 550 nm, which is clearly seen in the ‘flux OUT’ in Fig. 5d. However, differently, if the pump pulse is immediately followed by a STED pulse of RHC-polarized light with sufficient energy and in resonance with one of the edge modes (in this case R3 in Fig. 5e), such a pulse is resonantly amplified via stimulated emission, leaving behind the dye mostly in its ground state. Owing to the depletion of the excited states, there is no energy available for lasing into the R1 mode at 550 nm. The lasing in the R1 mode is therefore suppressed and only the amplified STED pulse is radiated from the CLC microlaser at another mode, in our simulated case the R3 mode, as seen in Fig. 5e. Figure 5f shows the suppression of the lasing upon increasing the energy of the STED pulse in resonance with the R3 mode. We observe that the STED pulse is most effective when it is in resonance with one of the edge R modes (Fig. 5g). Excluding the R1 mode, where the lasing is observed, the lowest STED energy required for lasing to be turned off is at the R2 mode, followed by the R3 mode and so on. Furthermore, resonant STED is polarization sensitive. For example, the suppression of lasing is less effective if an equally strong pulse at the same wavelength is used, but has a handedness opposite to the helix. Such a left-handed circularly polarized STED pulse will only make an effective single pass through the right-handed CLC structure, not depleting enough excited dye, so some lasing is still detected in the spectrum (Extended Data Fig. 4).

## Conclusion

We demonstrate the successful integration of LC microlasers in polymer scaffolds on glass that are mutually interconnected with laser-printed polymer waveguides with very low propagation losses. The LC microlasers are ignited by nanosecond light pulses, and they emit nanosecond laser pulses at a wavelength that is tunable by LC composition. Most remarkably, we introduce the concept of resonant STED, which enables switching on and off of light signals from the integrated LC microlasers by light. The concept is based on stimulated emission from an optical microcavity that is in an excited state, thus providing optical gain for the light in the cavity. With no external photons being inputted, the electromagnetic field in the cavity spontaneously amplifies the light mode with the lowest losses. However, with external photons that are in resonance with one selected cavity mode, the energy that is stored in the excited cavity will be released by amplifying those external photons, which is akin to the well-known STED effect used in super-resolution imaging. Notably, the energy needed for STED in resonant microcavities is only a fraction of the energy needed to excite the lasing. This is in sharp contrast to single-pass STED used in super-resolution imaging, where the energy of the STED pulse is several orders of magnitude higher compared with the excitation pulse. Although in our experiments STED photons and lasing photons propagate in the same direction, defined by the helix of the CLC, we could imagine a large variety of configurations and geometries, where these photons travel in different directions in space.

The implemented soft-matter photonic integrated platform uses the self-assembly properties of chiral LCs to spontaneously form laser microcavities, thus reducing the number of steps to produce a laser by orders of magnitude compared with solid-state lasers. Moreover, this photonic integrated platform is based on nanosecond all-optic control of the flow of photons using different eigenmodes of optical microcavities to redirect the photons in space and time. This platform has the potential to realize chiral microreflectors, chiral microgratings, dye micropolarizers, optical microamplifiers and polarization-sensitive filters that could be controlled by light or electricity, to mention just a few. These active and passive microphotonic devices could be integrated into soft-matter integrated circuits akin to modern logic architectures, where microlasers are connected to each other and perform logic operations by feeding each other’s input via the STED effect. The platform could further benefit from exploring the novel design of CLC microcavities, such as a three-dimensional spherical CLC microcavity^34^, which offers omnidirectional access as well as interaction and control of a multitude of light beams in three dimensions. One could envisage other geometries of CLC microcavities that cannot be produced in hard matter, such as a square, box-like CLC cavity, cylindrical and so on. Finally, we should mention that soft nanoimprint lithographic replication^35^ could be used to produce a large number of complex, polymer-scaffold soft-matter chips by simply replicating the master chip. The presented results clearly demonstrate the potential of LCs to implement all-optic LC photonic integrated circuits that can be made both biodegradable and biocompatible with a rich variety of applications in medicine, wearable photonics and logic circuits.

## Methods

### DLW printing of waveguides and scaffolds

We used a two-photon lithography system (Photonic Professional GT+, Nanoscribe), which allows for a lateral resolution of down to 120 nm. The scaffolds and waveguides were printed on 22 mm × 22 mm borosilicate coverslips (ROTH) that were 170 μm ± 5 μm thick. The coverslips were first wiped with lint-free cloths and acetone, followed by a 15 min ultrasonic bath in a 1% water solution of Hellmanex II detergent at 35 °C. After another 15 min ultrasonic bath in pure deionized water to remove all traces of the detergent, the substrates were dried in an isopropanol vapour degreaser. The coverslips were then coated with a fluorinated polymer CYTOP (CTX-809A, Asahi Glass) that has a refractive index of 1.34 in the visible range. A 60:40 wt% solution of CTX-809A and solvent CT-SOLV9AP was spin-coated at 1,000 r.p.m. onto cleaned substrates and baked at 205 °C for 1 h. This process produced a fluoropolymer film of approximately 500 nm thickness that served as a low-refractive index optical isolation layer between the borosilicate glass and the DLW-printed polymer waveguides and scaffolds. To aid adhesion of the printed structures, the CYTOP-coated substrates were placed for 5 min into an ultraviolet ozone (UVO) cleaner (UVO-Cleaner 256-220, Jelight). After 5 min, the substrates were blown with nitrogen and a small amount of IPS resin (Nanoscribe) was drop-cast onto the substrate. The refractive index of non-cured IPS resin in the visible range is 1.486 (590 nm); after polymerization the refractive index was 1.515 (at 590 nm). A ×63 objective numerical aperture (NA; Zeiss, 1.4 NA Oil Dic, Plan Polychromat) was inserted into the PPGT+ microscope. The substrates were then loaded onto the substrate holder, which was then inserted into the machine with the resin side facing down to the objective lens. The resin acts as an immersion liquid; thus, this type of printing is called dip-in laser lithography. The objective was moved towards the substrate, and due to the refractive index mismatch of IPS and the substrate (>0.2), the interface was found automatically. After printing, the substrates were dipped twice, first for 12 min in PGMEA solvent (Sigma Aldrich) and second for 1 min in isopropanol. Finally, the substrates with printed structures are dried at 40 °C for 30 min.

### Measurement of propagation losses in DLW-printed waveguides

We used the standard cut-back method to measure propagation losses in DLW-printed optical waveguides in the visible range. We printed 6 waveguides with different lengths ranging from 150 µm to 900 µm in a single printing run. Each waveguide consists of two 90° prisms on each side and the rectangular waveguide with 10 × 10 µm cross-section. We used a tunable laser (Opolette UX10230, Opotek) as a light source, which was focused on the input prism using a ×10 objective (Nikon Plan Fluor, 0.30 NA) of an inverted microscope (Nikon Ti). The diameter of the input beam was larger than the cross-section of the prism to excite all waveguide modes. After the light propagates distance *L* along the waveguide, it is reflected by the output prism towards the objective of the microscope. The image of this light beam is then taken by a digital camera (FLIR Blackfly-U3-80S5C-C). The camera is focused on the bottom plane of the output prism, while the lateral position of the laser beam is optimized to the maximal intensity of light reflected out from the waveguide. For a given wavelength of light and length *L* of the waveguide, the output intensity *I* is plotted for each wavelength as a function of the waveguide length and fitted to the exponential function, to obtain the propagation loss value in dB cm^−1^ as presented in Fig. 1e. Propagation losses were measured for five different wavelengths of the input laser beam. In additional experiments, the intensity of the output light was measured with a spectrometer (Andor, SR500i) where the output light was collected using optical fibre and guided to the spectrometer. Both methods give comparable results for the propagation loss. The coupling loss of a single microprism was measured by comparing the intensity of the reflected beam from the mirror and the intensity of light transmitted through a very short waveguide with two microprisms. In this case, a ×20 objective (Nikon Plan Fluor, 0.50 NA) was used for better focusing of light on the microprism.

### SEM of waveguides and scaffolds

For control purposes, some of the printed waveguides and scaffolds hosting the LC were imaged using a scanning electron microscope. The substrates with the printed structures were cut to the appropriate size for imaging and coated with 10 nm of pure gold. The imaging was performed using a field-emission SEM Zeiss SUPRA 35 VP, using secondary electrons to reveal the topography of the imaged structures. The printed structures were imaged either from the top or tilted by 30° to obtain the correlative side view as well. For high-resolution SEM imaging, a Thermo Fisher Verios 4G HP Schottky field-emission SEM with monochromator was used. The sample preparation for imaging was the same until the last step, where the samples were coated with 8 nm of a gold/palladium mixture.

### Surface roughness of waveguides as measured by atomic force microscopy

We used atomic force microscopy to measure the surface roughness of the DLW-printed waveguides. The topography of the surface was obtained with a Nanoscope IIIa Multimode scanning probe microscope (Digital Instruments) operating in tapping mode using standard tapping mode probes OTESPA (Mikromasch) with a tip radius below 7 nm and a nominal resonant frequency of 300 kHz. Several images of different IPS waveguides were analysed and the average RMS surface roughness was 2.1 ± 0.3 nm (Extended Data Fig. 1).

### Preparation of LC mixtures and cells

To prepare the high-birefringence CLC mixture with R1 mode at 550 nm, a 3.74 wt% chiral dopant R5011 was mixed with the high-birefringence (Δ*n* ≈ 0.405) nematic LC GCHC10146 (Qingdao Grand International) in a small glass vial. The mixture was heated above the isotropic phase of both mixtures (~150 °C) on a hotplate and stirred well using a vortex mixer. After approximately 5 min, the sample was gradually cooled to room temperature. A 1 wt% fluorescent-dye (PM580) solution was prepared in acetone at room temperature and mixed well to ensure that all the dye was dissolved. To prepare the dye-doped CLC mixture, the prepared acetone–dye solution was mixed with the CLC in a weight ratio resulting in a 0.2 wt% dye concentration in the CLC–dye mixture. To evaporate all the acetone out of the solution, the bottle with the sample was placed in a ventilated oven at 60 °C for 24 h.

The cells containing the CLC were constructed using glass pieces of desired sizes. Before cell preparation, both glass plates were coated with a thin (~20 nm) layer of polyimide (SE-5291, Nissan) and rubbed uniformly using a velvet cloth to ensure homogeneous alignment of the CLC molecules with respect to the glass substrate. The glasses were stacked with antiparallel rubbing to each other, and the separation between the inner surfaces of the 2 glass plates was ensured using 9 μm silica beads mixed in ultraviolet glue. The glue was applied to several spots on glass around the cell boundaries and low pressure was applied to the cell to ensure the desired cell gap. The glue was then cured under ultraviolet light for about 2 min. The cell thickness between 8 μm and 9 μm was determined by measuring the interference pattern before filling the cell. The CLC was filled into the cell via capillary force at room temperature. Once completely filled, the cell was heated on a hotplate to about 60 °C for up to 24 h, during which time the CLC structure slowly relaxed and became uniform.

### Filling the scaffolds with LCs

An LC mixture, typically doped with a fluorescent dye, was injected into the printed scaffold using capillary force. We used a microinjector (FemtoJet 4i, Eppendorf) and a glass micropipette with a 1.0 µm outer diameter (Femtotip, Eppendorf). The filling was done by placing the end of the micropipette close to the inside wall of the polymer scaffold. As the LC wets the polymer walls very well, it is drawn out of the pipette by capillary force, thus slowly filling the scaffold. To aid the alignment of the LC and minimize the formation of topological defects inside the printed scaffolds, the filling was done slowly. Typically, the filling time for a single scaffold was kept in the range of 30 s to 1 min at an injection pressure of less than 100 kPa. The planar alignment of the LC was ensured by grooves on the scaffold surfaces, which result from the intensity variations of the laser light during the printing process. After filling, the CLC the structures were left to relax at an elevated temperature of 40 °C for 24 h.

### Polarizing microscopy of cells and microstructures

The samples were observed and imaged using a Nikon Ti2 Eclipse microscope equipped with an S Plan Fluor ×20 (0.45 NA) objective with a glass thickness correction collar. For optimal observation of cholesteric planar cells and maximum contrast in their transmission spectra, a right-handed circular polarizer, consisting of a polymer polarizer and a broadband quarter-wavelength retarder (American Polarizers) was inserted between the illumination source and the sample. For measurements of transmission spectra within the scaffolds, we used light from a supercontinuum laser (Fianium SC-450-PP-HE-09). The light was coupled into the fibre and fed to the microscope through the same back port as the beam used for STED (Extended Data Fig. 2, white light beam is not shown). The light was reflected from the plate beam splitter through the objective and illuminated one of the printed coupling prisms. When further increase of contrast was needed, another right-handed circular polarizer was inserted as an analyser, so that only RHC-polarized light was transmitted to the optical fibre and camera.

### Set-up for lasing and STED experiments

The optical set-up used for the STED experiments is shown in Extended Data Fig. 2. In a typical experiment, we used light from a nanosecond pulsed laser Opotek Opolette UX21040, which produces multiple outgoing beams. We used two of those outputs, namely, the 532 nm excitation beam, which is also used as one of the pump beams for the optical parametric oscillator that produces a wavelength tunable output that was used for STED. The outputs produce ~5–10 ns full-width at half-maximum (FWHM) pulses for excitation and STED pulses, respectively, at a maximum frequency of 20 Hz. The excitation pulse exits the laser before the STED pulse and for this reason needs to be delayed, which is achieved using a delay line with an additional optical path and a retroreflector, marked with R1 in Extended Data Fig. 2. The delay between the two pulses is set so that they arrive roughly simultaneously, which proved optimal in the later measurements. For both beams, two lenses (L1–L4) in a ‘4*f*’ configuration were used in combination with carefully positioned mirrors (M1–M8) on the optical table to enable independent beam positioning in the plane of the sample. To control the energy of the pulses hitting the sample, we used a combination of rotatable and fixed polarizers. The rotatable polarizer was controlled via computer software, which enabled precise and repeatable energy settings. Finally, to vary the light polarization circularity at the sample plane, we used 532 nm and achromatic rotatable quarter waveplates, for excitation and the STED beam, respectively, positioned before the back entrance of the microscope. Inside the microscope, the beams were directed towards the sample using plate beam splitters. To cut the back-reflecting excitation light, a 532 ± 10 nm band-stop filter (optical density 6) was used. The remaining light was led to the side exit port of the microscope where exactly in the image plane of the microscope a rectangular mirror was placed, at an angle of 45°. The central pinhole in the mirror had a diameter of 500 μm and allowed the light from a particular position within the image to pass through. There it was coupled into a 105 μm core multimode fibre and led to the fibre-input port of the spectrometer Andor SR500i equipped with a Andor Newton EMCCD camera. Spectra were measured using a 1200 l/mm grating, which provided a resolution of at least 0.11 nm, measured using a 632.8 nm HeNe laser. The light reflected from the pinhole mirror passed through a matched lens pair (*f* = 50 mm), positioned in a way that the image in the microscope image plane was relayed onto the sensor of the Flir BFS-U3-50S5C camera. The mirror–camera–fibre set-up was placed on an *xyz* translation stage, which allowed for precise positioning of the pinhole with respect to the microscope image and with that the position of spectral measurement.

Before or after performing measurements on the microlaser structures, the sample was replaced by an energy meter Ophir PD10-pJ-C and set to the appropriate wavelength. The energy of both beams was then measured for different orientations of the rotatable polarizer. To decrease the influence of pulse-to-pulse energy fluctuations inherent to the pulsed laser source, 100 pulses were acquired for each angle setting and their energies were averaged. To calculate the corresponding energy fluences, images of involved beam spots in the sample plane were taken. From the radial profiles of the nearly circular beam spots, an average FWHM beam radius was determined and used to calculate the beam fluences from the recorded beam energies.

### Numerical simulations of CLC microlasers and resonant STED

Light propagation with saturable absorption and stimulated emission was numerically modelled in the software tool MEEP^25^ appropriately adapted and used for simulations of anisotropic CLC optical media. It is based on the finite-difference time-domain method^26^, which propagates electric (**E**) and magnetic fields (**H**) in space (**r** = (*x*,*y*)) and time (*t*)—**E**(**r**, *t*) and **H**(**r**, *t*)—according to full Maxwell’s equations in a given geometry described by the dielectric tensor field $\underline{\varepsilon }({\bf{r}})$ and field sources.

#### Geometry and materials

In isotropic materials with refractive index *n*, the dielectric tensor is ${\varepsilon }_{{ij}}={n}^{2}{\delta }_{{ij}}$, whereas in anisotropic LCs with ordinary refractive index *n*_o_ and extraordinary refractive index *n*_e_, and director field ${\bf{n}}=\left({n}_{x},{n}_{y},{n}_{z}\right)$, it becomes ${\varepsilon }_{{ij}}={n}_{o}^{2}{\delta }_{{ij}}+\left({n}_{{\mathrm{e}}}^{2}-{n}_{o}^{2}\right){n}_{i}{n}_{j}$, where δ_*ij*_ is Kronecker delta and $i,j\in \{x,y,z\}$. The studied effectively two-dimensional dielectric structure with labelled dimensions and refractive indices is shown in Extended Data Fig. 3a.

The director field of the right-handed CLC with helical axis along the *y* axis inside the cavity is parameterized as ${\bf{n}}=(\cos \frac{2\uppi y}{p},0,-\sin \frac{2\uppi y}{p})$, where *p* is the cholesteric pitch. The whole structure is surrounded by perfectly matched layers, which serve as perfect absorbers to provide open boundary conditions.

Absorption and stimulated emission on organic dye molecules, where the emission and absorption spectra are shifted in wavelength, are described by a four-level system (Extended Data Fig. 3b). The emission and absorption spectra are compared with the transmittance of the CLC in Extended Data Fig. 3c. In the simulations in Meep, in addition to the electromagnetic fields, the fields of the occupations of energy levels ${N}_{1}\left({\bf{r}},t\right)$, ${N}_{2}({\bf{r}},t)$, ${N}_{3}({\bf{r}},t)$ and ${N}_{4}({\bf{r}},t)$ are also calculated in space and time. The coupling of the electric field with the absorption transition (a) between levels 1 and 4 with the spectrum centred at frequency *ω*_a_ and FWHM frequency width *γ*_a_, or with the stimulated-emission transition (e) between levels 2 and 3 with the spectrum centred at *ω*_e_ and FWHM width *γ*_e_, is described by the classical oscillator model for polarization **P**_a,e_(**r**) via:

$$\begin{array}{l}\displaystyle\frac{{{\rm{d}}}^{2}{{\bf{P}}}_{{\mathrm{a,e}}}({\bf{r}},t)}{{\rm{d}}{t}^{2}}+{\gamma }_{{\mathrm{a,e}}}\displaystyle\frac{{\rm{d}}{{\bf{P}}}_{{\mathrm{a,e}}}({\bf{r}},t)}{{\rm{d}}t}+\left({\omega }_{{\mathrm{a,e}}}^{2}+{\left(\displaystyle\frac{{\gamma }_{{\mathrm{a,e}}}}{2}\right)}^{2}\right){{\bf{P}}}_{{\mathrm{a,e}}}\left({\bf{r}},t\right)\\=-\varDelta {N}_{{\mathrm{a,e}}}\left({\bf{r}},t\right){\sigma }_{{\mathrm{a,e}}}{\bf{E}}\left({\bf{r}},t\right),\end{array}$$

where $\Delta {N}_{{\mathrm{a}}}({\bf{r}},t)={N}_{4}({\bf{r}},t)-{N}_{1}({\bf{r}},t)$, $\Delta {N}_{{\mathrm{e}}}({\bf{r}},t)={N}_{3}({\bf{r}},t)-{N}_{2}({\bf{r}},t)$, *σ*_a_ is the isotropic absorption cross-section and *σ*_e_ is isotropic stimulated-emission cross-section. Time-dependent polarization is then added to the electric displacement field (**D**) as ${\bf{E}}\left({\bf{r}},t\right)={\bf{D}}\left({\bf{r}},t\right)-{{\bf{P}}}_{{\mathrm{a}}}\left({\bf{r}},t\right)-{{\bf{P}}}_{{\mathrm{e}}}\left({\bf{r}},t\right)$ at each step of the simulation.

Owing to the described electric-field-polarization coupling in transitions (a) 1 → 4 and (e) 3 → 2, and owing to non-radiative (vibrational) transitions from level 4 to level 3 with selected rate *Γ*_43_ and from level 2 to level 1 with rate *Γ*_21_, the level populations change as^25^:

$$\frac{\partial {N}_{1}\left({\bf{r}},t\right)}{\partial t}=+{\varGamma }_{21}{N}_{2}\left({\bf{r}},t\right)-\frac{1}{{\omega }_{{\mathrm{a}}}{{\hslash }}}{\bf{E}}\left({\bf{r}},t\right)\cdot \left(\frac{\partial }{\partial t}+\frac{{\gamma }_{{\mathrm{a}}}}{2}\right){{\bf{P}}}_{{\mathrm{a}}}\left({\bf{r}},t\right),$$

$$\frac{\partial {N}_{2}\left({\bf{r}},t\right)}{\partial t}=-{\varGamma }_{21}{N}_{2}\left({\bf{r}},t\right)-\frac{1}{{\omega }_{{\mathrm{e}}}{{\hslash }}}{\bf{E}}\left({\bf{r}},t\right)\cdot \left(\frac{\partial }{\partial t}+\frac{{\gamma }_{{\mathrm{e}}}}{2}\right){{\bf{P}}}_{{\mathrm{e}}}\left({\bf{r}},t\right),$$

$$\frac{\partial {N}_{3}({\bf{r}},t)}{\partial t}=+{\varGamma }_{43}{N}_{4}\left({\bf{r}},t\right)+\frac{1}{{\omega }_{{\mathrm{e}}}{{\hslash }}}{\bf{E}}\left({\bf{r}},t\right)\cdot \left(\frac{\partial }{\partial t}+\frac{{\gamma }_{{\mathrm{e}}}}{2}\right){{\bf{P}}}_{{\mathrm{e}}}\left({\bf{r}},t\right),$$

$$\frac{\partial {N}_{4}({\bf{r}},t)}{\partial t}=-{\varGamma }_{43}{N}_{4}\left({\bf{r}},t\right)+\frac{1}{{\omega }_{{\mathrm{a}}}{{\hslash }}}{\bf{E}}\left({\bf{r}},t\right)\cdot \left(\frac{\partial }{\partial t}+\frac{{\gamma }_{{\mathrm{a}}}}{2}\right){{\bf{P}}}_{{\mathrm{a}}}\left({\bf{r}},t\right).$$

To qualitatively correspond with experiments, the parameters are set to the following values (in units of 2π*c*_0_ μm^−1^, where *c*_0_ is the speed of light in vacuum): ${\omega }_{{\mathrm{a}}}=1/{\lambda }_{{\mathrm{a}}}=1/(0.530)$, ${\omega }_{{\mathrm{e}}}=1/{\lambda }_{{\mathrm{e}}}=1/(0.550)$ and ${\gamma }_{{\mathrm{a}}}={\gamma }_{{\mathrm{e}}}=0.13$. In particular, these values fit the absorption and emission spectra of the dye used in experiments centred at approximately 530 nm and 550 nm, respectively, and with the FWHM of both spectra approximately 40 nm. Absorption and stimulated-emission cross-sections are set to *σ*_a_ = *σ*_e_ = 0.006, and transition rates (in units of *c*_0_ μm^−1^) to *Γ*_43_ = 10, *Γ*_21_ = 100. Initially, the dye is everywhere in the ground state with ${N}_{1}({\bf{r}},t=0)=25$, ${N}_{2}({\bf{r}},t=0)=0$, ${N}_{3}({\bf{r}},t=0)=0$ and ${N}_{4}({\bf{r}},t=0)=0$, which then changes locally due to pump, STED and lasing.

#### Sources of electromagnetic waves

Both pump and STED pulses, which propagate through the studied structure, originate in a waveguide at a distance of 1.6 μm from the simulation domain boundary. The width of the source area is 5 μm, and it is equal to the width of the waveguide, and the source produces fields in the shape of a Gaussian beam with focus at the source surface and with a waist size 2 μm. The time envelopes of both pulses are set to Gaussian profiles with a FWHM of 1,309 fs (393 Meep units). The vacuum wavelength of the pump pulse is 530 nm, and 561.2 nm for the STED pulse for the results shown in Fig. 5a–f, whereas in Fig. 5g, the STED wavelength is also varied. The time delay between the pump pulse and the STED pulse is 2,000 fs (600 Meep units). The amplitudes of sources are also varied but, in the simulations, resulting in Fig. 5a–e, they are set to dimensionless values between 0 and $\sqrt{32}$ (strongest pump pulse in Fig. 5f).

A ‘seed’ planar source of width 3 μm (generating weak radiation mostly in the +*y* and −*y* directions) with very small dimensionless amplitude (0.0002) and broad spectrum (centred at 530 nm and with FWHM 143 nm) is used to initiate lasing, and it placed in the centre of the cavity. The use of the seed source is necessary as the above explained classical description of lasing cannot simulate spontaneous emission. It turns on and off every 167 fs with alternating orientation of linear polarization: odd pulses are polarized along the *x* axis and even pulses are polarized along the *z* axis. Most of this low-intensity light only propagates out of the cavity, whereas the fields with frequencies corresponding to the eigen-frequencies of the cavity are effectively trapped inside the cavity and amplify to lasing.

#### Calculation of observable quantities

Throughout the simulation, the intensity flux (surface integral of the Poynting vector) is accumulated in the area labelled ‘flux IN’ and ‘flux OUT’ in Extended Data Fig. 3a, as implemented in Meep. The spectrum of the accumulated energy of light propagating through this surface by time *t* is labelled as $\varPhi (\lambda ,t)$ and is calculated via Fourier transformation. Spectra $\varPhi \left(\lambda ,T\right)$, where *T* is the total simulation time, are shown in Fig. 5d,e. The current flux is calculated as a time derivative $\phi \left(\lambda ,t\right)={\rm{d}}\varPhi \left(\lambda ,t\right)/{\rm{d}}t$. The current ‘integrated energy fluxes’ of the pump/STED/lasing pulses with finite spectral width, which are shown in Fig. 5b, are calculated as integrals $P\left(t\right)[{\lambda }_{1},{\lambda }_{2}]={\int }_{{\lambda }_{1}}^{{\lambda }_{2}}\phi \left(\lambda ,t\right){\rm{d}}\lambda$, where $\left[{\lambda }_{1},{\lambda }_{2}\right]=[527.5\,{\rm{nm}},\,532.5\,{\rm{nm}}]$ for the pump pulse, $\left[{\lambda }_{1},{\lambda }_{2}\right]=[559.2\,{\rm{nm}},\,564.9\,{\rm{nm}}]$ for the STED pulse and $\left[{\lambda }_{1},{\lambda }_{2}\right]=[547.3\,{\rm{nm}},\,552.7\,{\rm{nm}}]$ for the lasing pulse. When changing the wavelength of the STED pulse (Fig. 5f), this interval is also shifted accordingly. In Fig. 5b, the flux of STED versus pump pulse is multiplied by a factor of 25 and the lasing pulse by a factor of 200 for visual clarity, as the magnitudes of the pulses are different. The total energies of pulses, which are shown in Fig. 5f,g are calculated as $E[{\lambda }_{1},{\lambda }_{2}]={\int }_{\!{\lambda }_{1}}^{{\lambda }_{2}}{\int }_{\!0}^{T}\phi \left(\lambda ,t\right){\rm{d}}t{\rm{d}}\lambda$, where *T* is the total simulation time. Fluxes that are marked as ‘flux IN’ are calculated only on a straight waveguide without a cavity, which avoids numerical method caused reflections and improves accuracy.

#### Resonant and non-resonant STED

STED pulses that are circularly polarized with the same handedness as the structural helix are more effective in turning off lasing than STED pulses with the opposite polarization. Resonant modes in right-handed CLCs exist for only RHC-polarized light. An RHC-polarized pulse at the edge mode frequencies is resonantly trapped in the sample, its group velocity is reduced, and it actually passes the optical gain material several times to more effectively deplete the population inversion in the optical gain material.

At a selected intensity of the STED pulse that operates at the R3 mode, 562.1 nm, the lasing in the R1 mode (550 nm) is effectively turned off by an RHC-polarized pulse, as shown in Extended Data Fig. 4a, which is identical to Fig. 5e. On the contrary, if the STED pulse with equal intensity is left circularly polarized (Extended Data Fig. 4b), the pulse makes only a single pass through the optical gain material. Therefore, it is only slightly amplified (compare STED IN and STED OUT for both polarizations), and does not sufficiently deplete the inversion in the gain material, allowing lasing to still develop at 550 nm, which is shown in green in Extended Data Fig. 3b.

### Reporting summary

Further information on research design is available in the Nature Portfolio Reporting Summary linked to this article.

## Online content

Any methods, additional references, Nature Portfolio reporting summaries, source data, extended data, supplementary information, acknowledgements, peer review information; details of author contributions and competing interests; and statements of data and code availability are available at 10.1038/s41566-025-01693-2.

## Supplementary information

Supplementary Information Supplementary Fig. 1 and Discussion.

Reporting Summary

Supplementary Video 1 Printing of IPS polymer, U-shaped waveguide. The waveguide is printed onto low-refractive-index CYTOP-coated coverslips using dip-in laser lithography mode, with IPS resin as the immersion liquid. The waveguide is constructed layer by layer, with a layer spacing of 100 nm. At each layer, the structure is formed by guiding the laser focus in progressively smaller contours using galvo mirrors. The distance between two successive contour lines is set to 100 nm and the laser is moved at a speed of 1,000 μm s^−1^. The typical print time for a U-shaped waveguide with a length of 300 μm and a 10 μm square cross-section is approximately 45 min. The video is sped-up 300 times.

Supplementary Video 2 Transmission of visible light through DLW-printed waveguides. The video shows transmission of visible light through 100-μm-long rectangular waveguides as their entry prism (top of the frame) is moved continuously across the input beam in a lateral direction. It can be seen how the coupling efficiency is strongly position dependent. The IPS fibres were printed using DLW on a thin layer of CYTOP polymer coated on glass. Several visible light wavelengths from blue to red are shown and their values are noted in the top-right corner of each video inset.

Supplementary Video 3 Printing of IPS polymer scaffold hosting five microlasers with various waveguide size. The polymer scaffold is printed onto CYTOP-coated coverslips using dip-in laser lithography mode, with IPS resin as the immersion liquid. The scaffold is constructed layer by layer, with a layer spacing of 100 nm. At each layer, the structure is formed by tracing progressively smaller contours with the laser focus using galvo mirrors. The distance between two successive contour lines is set to 100 nm and the laser is moved at a speed of 1,000 μm s^−1^. A typical print time for one structure is approximately 1 hour. The video is sped-up 400 times.

Supplementary Video 4 Filling the IPS polymer scaffold with an LC. A micropipette filled with an LC mixture (GCHC10146 + R5011) is being lowered into the 8.2 μm printed laser cavity onto which 10 μm microprisms and waveguides are connected. As the tip of the micropipette touches the wall of the scaffold, the LC is drawn out of the pipette due to capillary force. Typically, the filling of the scaffolds is done slowly to ensure good planar alignment of the LC inside the resonator cavity. The quality of the alignment is indicated by the homogeneity of the LC texture inside the scaffold. The video shows a typical filling process, which takes 45 s. The video is sped-up 5 times.

Supplementary Video 5 Printing of the smallest microlaser IPS scaffold with a 1-μm-thick waveguide. The scaffold is printed onto CYTOP-coated coverslips using dip-in laser lithography mode, with IPS as the immersion liquid. The scaffold is constructed layer by layer, with a layer spacing of 100 nm. At each layer, the structure is formed by tracing progressively smaller contours with the laser focus using galvo mirrors. The distance between two successive contour lines is set to 100 nm and the laser is moved at a speed of 1,000 μm s^−1^. A typical print time for one structure is approximately 30 min. The video is sped-up 200 times.

Supplementary Video 6 A dynamic rotation SEM view of the smallest footprint microlaser. This video was produced by taking 36 individual SEM images at different rotation angles of the printed structures. Additional SEM snapshots were generated using artificial intelligence and all the frames were incorporated into a video, giving the impression of smooth, dynamic rotation.

Supplementary Video 7 Lasing of the smallest footprint microlaser. The video shows pulsed lasing of a microstructure laser with 1 μm fibres, filled with a CLC–dye mixture lasing at 553 nm. The left-hand side of the frame shows the acquired spectrum of each individual lasing pulse and the right-hand side shows a photograph of the structure in that same moment. The light signal taken to the spectrometer was acquired from the top microlaser prism as is apparent from the pinhole position (dark circle). The microlaser was pumped through the same prism with a constant energy fluence *I*_EXC_ noted above the spectrum.

Supplementary Video 8 Lasing threshold of the smallest footprint microlaser. The video shows the onset of lasing in a CLC-filled microstructure with 1 μm fibres that occurs by increasing the pump energy across the lasing threshold at pump fluence of ∼85 pJ μm^−2^. The left-hand side of the frame shows the acquired spectrum of each individual pulse and the right-hand side shows a photograph of the structure in that same moment. The light signal taken to the spectrometer was acquired from the top microlaser prism, as is apparent from the pinhole position (dark circle). The microlaser was pumped through the same prism with a variable energy fluence *I*_EXC_ noted above each spectrum.

Supplementary Video 9 Illustration of lasing and lasing depletion in CLC microlasers.

Supplementary Video 10 Switching the lasing off by using near-resonant STED. The video shows how lasing at ~550 nm is gradually switched off by increasing the energy of a simultaneous STED beam at 553 nm. The left-hand side of the frame shows the acquired spectrum of each individual lasing pulse and the right-hand side shows a photograph of the structure in that same moment. The increasingly brighter green spot is the 553 nm STED beam that is partially reflected from the CLC structure. The light signal taken to the spectrometer was acquired from the bottom microlaser prism as is apparent from the pinhole position (dark circle). The microlaser was pumped through the top prism with a fixed energy fluence *I*_EXC_, while the STED beam energy fluence *I*_STED_ was increased gradually. Values of both are noted above each spectrum.

Supplementary Video 11 Switching the lasing off by using far-detuned STED. The video shows control of lasing output from a microlaser structure. The left-hand side of the frame shows the acquired spectrum of each individual pulse and the right-hand side shows a photograph of the structure in that same moment. Initially operating laser is switched off at frame 38, by switching on a far-detuned STED beam at 603 nm, marked on the spectrum with a red vertical band. After about 40 frames (2 s) the STED beam is switched off again and lasing reappears. To make the observation of the effect using a camera possible, the strong STED beam was filtered using a short-pass filter at 600 nm, also causing the greener appearance of the video background.

Supplementary Video 12 Numerically simulated time evolution of light pulses in the CLC cavity in lasing and STED regimes. The video shows the time evolution of the |*E*_*z*_(*t*)| component of the electric field in the two regimes—lasing and STED. On the left is the lasing regime and on the right is the STED regime. Time runs synchronously in both parts of the video. In both cases, the optical gain material in the right-handed CLC is first locally pumped to the excited state with left circularly polarized light of 530 nm wavelength (light blue). On the right, the pump pulse is immediately followed by a resonant STED pulse (orange), which has a right circular polarization and a wavelength corresponding to the R3 mode at 562.1 nm. On the left, there is no emergent STED pulse, but lasing appears, whereas on the right due to resonant STED-caused reduced inversion in the optical gain no lasing appears.

## Data Availability

Most of the materials are commercially available, whereas some materials are subject to materials transfer agreements. All data are available in the article and its Supplementary Information and via Zenodo at 10.5281/zenodo.15025493 (ref. ^36^).

## Code availability

The code used for printing microstructures is available upon reasonable request.

## References

[1] de Gennes, P. G. & Prost, J. *The Physics of Liquid Crystals* (Clarendon Press, 1993).

[2] Khoo, I.-C. & Wu, S.-T. *Optics and Nonlinear Optics of Liquid Crystals* (World Scientific, 1993).

[3] Lueder, E. *Liquid Crystal Displays* (John Wiley & Sons, 2010).

[4] Kopp, V. I., Fan, B., Vithana, H. K. M. & Genack, A. Z. Low-threshold lasing at the edge of a photonic stop band in cholesteric liquid crystals. *Opt. Lett.* **23**, 1707–1709 (1998). 18091891 10.1364/ol.23.001707

[5] Coles, H. & Morris, S. Liquid-crystal lasers. *Nat. Photon.* **4**, 676–685 (2010).

[6] Sultanov, V. et al. Tunable entangled photon-pair generation in a liquid crystal. *Nature* **631**, 294–299 (2024). 38867054 10.1038/s41586-024-07543-5 PMC11236711

[7] Deubel, M. et al. Direct laser writing of three-dimensional photonic-crystal templates for telecommunications. *Nat. Mater.* **3**, 444–447 (2004). 15195083 10.1038/nmat1155

[8] Nocentini, S. et al. Three-dimensional photonic circuits in rigid and soft polymers tunable by light. *ACS Photon.* **5**, 3222–3230 (2018).

[9] Lee, C. H., Yoshida, H., Miura, Y., Fujii, A. & Ozaki, M. Local liquid crystal alignment on patterned micrograting structures photofabricated by two photon excitation direct laser writing. *Appl. Phys. Lett.* **93**, 173509-1–3 (2008).

[10] Ji, Z. et al. Compartmentalized liquid crystal alignment induced by sparse polymer ribbons with surface relief gratings. *Opt. Lett.* **41**, 336–339 (2016). 26766708 10.1364/OL.41.000336

[11] He, Z., Tan, G., Chanda, D. & Wu, S.-T. Novel liquid crystal photonic devices enabled by two-photon polymerization. *Opt. Exp.* **27**, 11472–11491 (2019). 10.1364/OE.27.011472 31052991

[12] Shi, Y. et al. Two-photon laser-written photoalignment layers for patterning liquid crystalline conjugated polymer orientation. *Adv. Funct. Mater.* **31**, 2007493 (2021).

[13] O’Neill, J. S. et al. 3D switchable diffractive optical elements fabricated with two-photon polymerization. *Adv. Optical Mater.* **10**, 2102446 (2022).

[14] Martinez, A. et al. Mutually tangled colloidal knots and induced defect loops in nematic fields. *Nat. Mater.* **13**, 258–263 (2014). 24390381 10.1038/nmat3840

[15] Yuan, Y., Martinez, A., Senyuk, B., Tasinkevych, M. & Smalyukh, I. I. Chiral liquid crystal colloids. *Nat. Mater.* **17**, 71–78 (2018). 29180773 10.1038/nmat5032

[16] Shi, Y. et al. Two-photon laser-written photoalignment layers for patterning liquid crystalline conjugated polymer orientation. *Adv. Funct. Mater.* **31**, 2007493-1–2007493-9 (2021).

[17] Nocentini, S., Martella, D., Parmeggiani, C., Zanotto, S. & Wiersma, D. S. Structured optical materials controlled by light. *Adv. Opt. Mater.* **6**, 1800167-1–1800167-9 (2018).

[18] O’Neill, J. S. et al. 3D switchable diffractive optical elements fabricated with two-photon polymerization. *Adv. Opt. Mater.* **10**, 2102446-1–10 (2022).

[19] Xiang, C. et al. 3D integration enables ultralow-noise isolator-free lasers in silicon photonics. *Nature* **620**, 78–84 (2023). 37532812 10.1038/s41586-023-06251-w PMC10396957

[20] Soref, R. A. & Lorenzo, J. P. All-silicon active and passive guided-wave components for *λ*=1.3 and 1.6 μm. *IEEE J. Quantum Electron.* **22**, 873–879 (1986).

[21] Soref, R. & Bennett, B. Electrooptical effects in silicon. *IEEE J. Quantum Electron.* **23**, 123–129 (1987).

[22] Almeida, V. R., Barrios, C. A., Panepucci, R. R. & Lipson, M. All-optical control of light on a silicon chip. *Nature* **431**, 1081–1084 (2004). 15510144 10.1038/nature02921

[23] Yeh, M.-P. et al. Characteristics of inorganic acid emission from various generation semiconductor manufacturing factories. *Chemosphere* **347**, 140745 (2024). 37981016 10.1016/j.chemosphere.2023.140745

[24] Ruberti, M. The chip manufacturing industry: environmental impacts and eco-efficiency analysis. *Sci. Total Environ.* **858**, 159873 (2023). 36334661 10.1016/j.scitotenv.2022.159873

[25] Hell, S. W. & Wichmann, J. Breaking the diffraction resolution limit by stimulated emission: stimulated-emission-depletion fluorescence microscopy. *Opt. Lett.* **19**, 780–782 (1994). 19844443 10.1364/ol.19.000780

[26] Hasan, M. & Blair, S. Maximizing transmittance in two-photon 3D printed materials for micro-optics in the visible. *Opt. Mater. Exp.* **12**, 895–906 (2022). 10.1364/ome.448819 PMC9386737 35993007

[27] Vellaichamy, M., Škarabot, M. & Muševič, I. Optical gain and photo-bleaching of organic dyes, quantum dots, perovskite nanoplatelets and nanodiamonds. *Liq. Cryst.* **50**, 935–956 (2023).

[28] Zaplotnik, J. et al. Photonic eigenmodes and transmittance of finite-length 1D cholesteric liquid crystal resonators. *Sci. Rep.* **13**, 16868 (2023). 37803161 10.1038/s41598-023-43912-2 PMC10558490

[29] Vitek, M. & Muševič, I. Nanosecond control and optical pulse shaping by stimulated emission depletion in a liquid crystal. *Opt. Exp.* **23**, 16921–16932 (2015). 10.1364/OE.23.016921 26191703

[30] Tai, J.-S. B. & Smalyukh, I. I. Super-resolution stimulated emission depletion microscopy of director structures in liquid crystals. *Opt. Lett.* **43**, 5158–5161 (2018). 30320844 10.1364/OL.43.005158

[31] Klar, T. A. & Hell, S. W. Sub-diffraction resolution in far-field fluorescence microscopy. *Opt. Lett.* **24**, 954–956 (1999). 18073907 10.1364/ol.24.000954

[32] Oskooi, A. F. et al. MEEP: a flexible free-software package for electromagnetic simulations by the FDTD method. *Comput. Phys. Commun.* **181**, 687–702 (2010).

[33] Taflove, A. & Hagness, S. C. *Computational Electrodynamics: The Finite-Difference Time-Domain Method* 3rd edn (Artech House, 2005).

[34] Humar, M. & Muševič, I. 3D microlasers from self-assembled cholesteric liquid-crystal microdroplets. *Opt. Express* **18**, 26995–27003 (2010). 21196976 10.1364/OE.18.026995

[35] Lin, R. & Rogers, J. M. Molecular-scale soft imprint lithography for alignment layers in liquid crystal devices. *Nano Lett.* **7**, 1613–1621 (2007). 17518505 10.1021/nl070559y

[36] Vellaichamy, M. et al. Raw data associated with the article “Microscale generation and control of nanosecond light by light in a liquid crystal”. *Zenodo*10.5281/zenodo.15025493 (2025). 10.1038/s41566-025-01693-2 PMC12226344 40621151

