# Supplementary material for: Microscale generation and control of nanosecond light by light in a liquid crystal
Source: Nat Photonics. 2025 Jun 3;19(7):758–66. doi: 10.1038/s41566-025-01693-2 (PMC12226344; doi:10.1038/s41566-025-01693-2)
Supplement: Supplementary file 1 — Supplementary Fig. 1 and Discussion. [file 41566_2025_1693_MOESM1_ESM.pdf]

nature photonics

Supplementary information

https://doi.org/10.1038/s41566-025-01693-2

Microscale generation and control of nanosecond light by light in a liquid crystal

In the format provided by the authors and unedited

Measurements of the Fluorescence, the Amplified Spontaneous Emission (ASE) and the Transition to Lasing in Planar CLC lasers

The PM580 fluorescent dye, mixed with our liquid-crystal (LC) samples, was excited using 532-nm, nanosecond, light pulses, which is close to the absorption maximum of the sample-dye mixture. Measurements of the dye emission in a pure nematic material revealed that the emission peak in the PM580-LC mixture is at ~ 545 nm, close to the red-band edge of the prepared chiral LC mixtures. These chiral LC mixtures were prepared by adding an appropriate mass of chiral dopant to the nematic LC to obtain the desired helical pitch and hence the desired band structure. The spectra in Figure S1 were acquired in a well-aligned, right-handed CLC mixed with the PM580 dye and filled in a planar cell of ~ 9 μm thickness – see Methods for further details on the preparation of this sample. A transparent dashed red line in Figure S1a shows the transmission spectrum of this planar CLC cell, corresponding to the right-hand y-axis of the graph. When a cholesteric mixture is well aligned within a cell or a micro-laser cavity, the smooth fluorescence spectrum that is observed in a pure, i.e., non-twisted, nematic, becomes modulated because of the band edge modes that are present in the helical structure of the CLC. This fluorescence spectrum is shown with black curves in Figure S1a. The ASE spectrum, taken through a Right-Handed Circular (RHC) polarizer is shown in a full, black curve. Above the lasing threshold, the lasing line clearly develops from the ASE RHC spectrum at a wavelength of 552 nm, marked with a green, vertical line. However, if the fluorescence is observed through a Left-Handed Circular (LHC) polarizer, the modulation of the fluorescence spectrum due to the band edge modes is absent, as can be seen from the black, dashed line in Figure S1a. The remaining broad signal detected using the LHC analyzer originates from the unpolarized

fluorescence of the sample. This indicates that both the ASE and the lasing are predominantly right-hand circularly polarized, which is in line with previous experiments on CLC lasers.

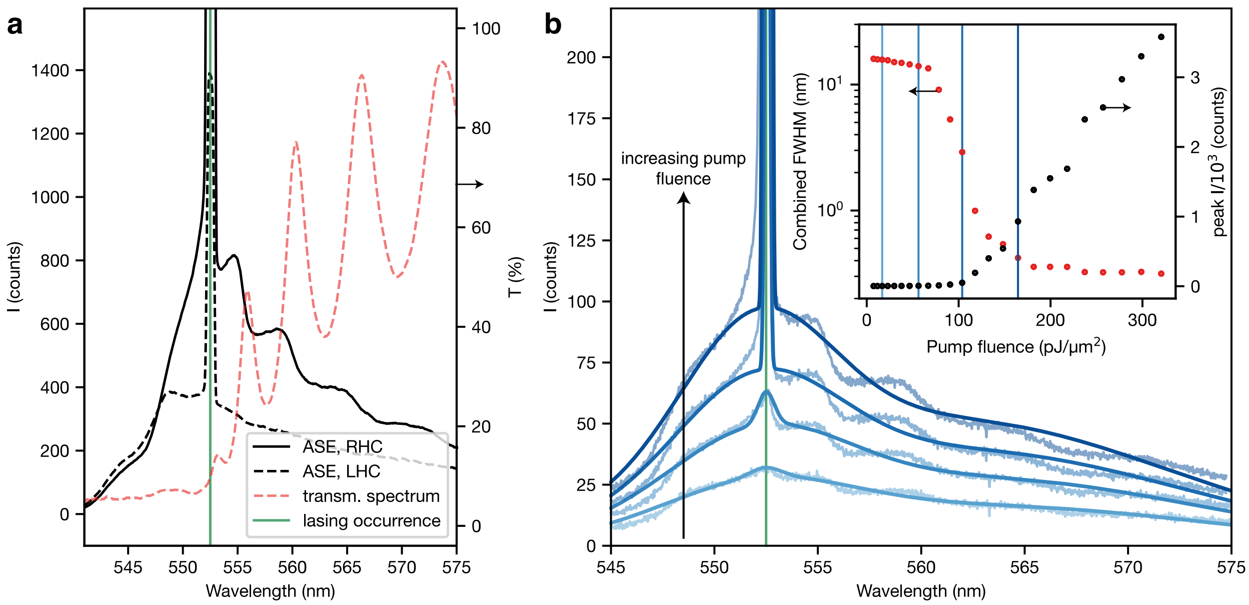

Figure S1. Lasing in planar CLCs. a Plot shows the lasing spectra of a right-handed, planar CLC laser pumped above the threshold, with the emitted light analyzed through left- (LHC) and right-handed circular (RHC) polarizers. The left-handed analyzer effectively blocks the ASE and most of the lasing signal, leaving only the broad contribution from unpolarized fluorescence. The red, dashed spectrum (right-hand axis) represents the transmission of the right-hand-polarized white light at the same position. **b** Emission spectra of a planar CLC laser at 4 increasing pump-fluence values. As the fluence increases, amplified spontaneous emission (ASE) peaks begin to emerge. Near the lasing threshold, the ASE peak at the red-band edge of the photonic bandgap (green vertical line) intensifies, culminating in lasing. Each spectrum is fitted using a combination of Gaussian curves laid over the spectra. The narrowing of the emission spectra is shown in the inset. The combined width of the Gaussian fit as a function of the pump fluence

clearly shows line narrowing as the lasing threshold is approached from below. The inset also shows a graph of the peak-intensity values (right-hand axis) as a function of the pump fluence, which indicates the lasing threshold value at $\sim 100\ \mathrm{pJ/\mu m^2}$.

Figure S1b shows the narrowing of the spectrum of a CLC laser with increasing pump fluence and the emergence of lasing. The spectra were fitted using a combination of three Gaussian-distribution curves, one for the main lasing peak and two for the broader fluorescence background contribution. The fitted curves are overlaid on the spectra with the corresponding fluence values shown using blue, vertical lines in the inset graph. The red markers in the inset graph show the combined full-width at half-maximum (FWHM) of the Gaussians of the form $g(\lambda) = ae^{-\frac{(\lambda-\lambda_0)^2}{2\sigma^2}}$ and correspond to the left-hand y-axis on a log scale. The combined width is defined as FWHM ~ $2.335\sigma_c$, where σ_c was calculated as an amplitude-weighted sum of the three Gaussian-distribution widths σ. Below a pump fluence of $\sim 60\ \mathrm{pJ/\mu m^2}$ only a very broad (FWHM ~ 15–20 nm) fluorescence is present. As the pump fluence increases, individual ASE peaks form (FWHM ~ 2 nm) and start to become sharper. At the lasing threshold, at ~ $100\ \mathrm{pJ/\mu m^2}$, the ASE peak closest to the transmission band edge increases very rapidly, forming a very narrow (FWHM ~ 0.2 nm) peak, characteristic of lasing. The right-hand axis corresponds to the peak (maximal) values of the spectra at different fluence values shown with black markers. We note that the combined FWHM value starts to reduce already at fluences below ~ $100\ \mathrm{pJ/\mu m^2}$, as the lasing peak starts to form, but its amplitude is not yet orders-of-magnitude above the fluorescence.
