# Supplementary material for: Microscale generation and control of nanosecond light by light in a liquid crystal
Source: Nat Photonics. 2025 Jun 3;19(7):758–66. doi: 10.1038/s41566-025-01693-2 (PMC12226344; doi:10.1038/s41566-025-01693-2)
Supplement: Supplementary file 2 — Reporting Summary [file 41566_2025_1693_MOESM2_ESM.pdf]

nature research

Corresponding author(s): Igor Muševič

Lasing Reporting Summary

Nature Research wishes to improve the reproducibility of the work that we publish. This form is intended for publication with all accepted papers reporting claims of lasing and provides structure for consistency and transparency in reporting. Some list items might not apply to an individual manuscript, but all fields must be completed for clarity.

For further information on Nature Research policies, including our data availability policy, see Authors & Referees.

Experimental design

Please check: are the following details reported in the manuscript?

1. Threshold

Plots of device output power versus pump power over a wide range of values indicating a clear threshold

☒ Yes
☐ No

The information can be found in Figure 2e for Direct Laser Writing (DLW) printed LC micro-lasers and in Figure 3j for the smallest operating CLC micro-laser.

2. Linewidth narrowing

Plots of spectral power density for the emission at pump powers below, around, and above the lasing threshold, indicating a clear linewidth narrowing at threshold

☒ Yes
☐ No

Plots of spectral output at different pump energies for a typical DLW printed micro-laser are shown in Figure 2d. Transition from the Amplified Spontaneous Emission to lasing and the line narrowing at threshold is presented in Supplementary Information.

Resolution of the spectrometer used to make spectral measurements

☒ Yes
☐ No

The spectrometer model and resolution are mentioned in "Methods" section in "Set up for lasing and STED experiments" section.

3. Coherent emission

Measurements of the coherence and/or polarization of the emission

☒ Yes
☐ No

Polarization properties of the emitted light were analyzed. Spectral measurements performed on the material confined in planar geometry confirm that above the lasing threshold the amplified spontaneous emission and lasing parts of the spectra pass through a broadband right-handed circular analyzer but are not transmitted by the left-handed circular analyzer, where only background fluorescence is detected. This indicates the lasing light has same sense of handedness as the helical CLC structure. This is mentioned in "Results and discussion" section when describing the chiral LC phase and explained in full detail in Supplementary Information.

4. Beam spatial profile

Image and/or measurement of the spatial shape and profile of the emission, showing a well-defined beam above threshold

☒ Yes
☐ No

Appearance of the spatial shape of light emitted from a DLW printed micro-laser is shown in the right inset of Figure 2e. Above the lasing threshold, the output light spatial profile shows characteristic speckles, presumably due to scattering and interference of the emitted lasing light within the material and DLW printed fibers.

5. Operating conditions

Description of the laser and pumping conditions
Continuous-wave, pulsed, temperature of operation

☒ Yes
☐ No

The method of fabricating the CLC dyed micro-lasers is well explained in the main text. The preparation of the DLW printed scaffolds and the cholesteric material containing the fluorescent dye are described in "Results and discussion" section and further expanded in "Methods". The pump conditions (nanosecond, pulsed, 532 nm) are described in the "Methods "section as well.

Threshold values provided as density values (e.g. W cm^{-2} or J cm^{-2}) taking into account the area of the device

☒ Yes
☐ No

Pump and STED beam fluences are stated throughout the text in J um^-2 units, where the beam cross-section area was taken into account and measured. This is mentioned in subsection "Set up for lasing and STED experiments" within the "Methods" section.

6. Alternative explanations

Reasoning as to why alternative explanations have been ruled out as responsible for the emission characteristics
e.g. amplified spontaneous, directional scattering; modification of fluorescence spectrum by the cavity

☒ Yes
☐ No

In "Results and discussion" section, during the discussion of Figure 2, we mention multiple points as of why the observed phenomenon is actually lasing: transition from fluorescence to ASE before the onset of actual lasing is clearly observed, clear distinction between featureless and speckled appearance of the output prism, below and above the threshold, respectively.

7. Theoretical analysis

Theoretical analysis that ensures that the experimental values measured are realistic and reasonable
e.g. laser threshold, linewidth, cavity gain-loss, efficiency

☐ Yes
☒ No

Simulations of lasing of a cholesteric LC confined to microstructures are presented, using a FDTD method, as described in section "Numerical simulations of chiral nematic micro-lasers and resonant STED" and further in the subsection "Numerical simulations of chiral nematic micro-lasers and resonant STED" within "Methods" section. However, the actual values of experimental values, e.g. lasing threshold, efficiency etc., cannot be determined, because of lack of some material parameters.

8. Statistics

Number of devices fabricated and tested

☐ Yes
☒ No

We designed, fabricated and tested more than 500 micro-lasers for this study. Several different designs of micro-laser devices were printed and tested, as shown in Figures 2 and 3. We were mainly dealing with the simplest scaffold type, shown in Figure 2, which showed reproducible results over a large number of tested scaffolds.

Statistical analysis of the device performance and lifetime (time to failure)

☐ Yes
☒ No

The device engineering is still in progress with an aim to increase the lifetime of micro-lasers by using inorganic optical gain materials. At present, we use organic dyes as optical gain material, which are susceptible to bleaching. The lifetimes and bleaching rates of dyes and inorganic gain materials for liquid crystal lasers have been presented in our article Vellaichamy et al. Optical gain and photo-bleaching of organic dyes, quantum dots, perovskite nanoplatelets and nanodiamonds, Liquid Crystals, 2023, DOI: 10.1080/02678292.2023.2188614.
